# Loss of SOX18/CLAUDIN5 disrupts the pulmonary endothelial barrier in ventilator-induced lung injury

**DOI:** 10.3389/fphys.2022.1066515

**Published:** 2022-12-22

**Authors:** Alejandro E. Garcia-Flores, Christine M. Gross, Evgeny A. Zemskov, Qing Lu, Kim Tieu, Ting Wang, Stephen M. Black

**Affiliations:** ^1^ Florida International University, Center for Translational Science, Miami, FL, United States; ^2^ Vascular Biology Center, Augusta University, Augusta, GA, United States; ^3^ Department of Medicine at Washington Hospital Center, Washington, DC, United States; ^4^ Department of Cellular Biology and Pharmacology, Herbert Wertheim College of Medicine Florida International University, Miami, FL, United States; ^5^ Department of Environmental Health Sciences, Robert Stempel College of Public Health and Social Work Florida International University, Miami, FL, United States

**Keywords:** mechanical stress, SOX-18, claudin-5, ALI, endothelial barrier function, tight junctions

## Abstract

Mechanical strain contributes to ventilator-induced lung injury (VILI) through multi-factorial and complex mechanisms that remain unresolved. Prevailing evidence suggests that the loss of pulmonary endothelial tight junctions (TJs) plays a critical role. TJs are dynamically regulated by physiologic and hemodynamic forces to stabilize the endothelial barrier. The transcription factor sex-determining region Y-box (SOX)-18 is important in regulating blood vessel development and vascular permeability through its ability to regulate the transcription of Claudin-5, an endothelial TJ protein. Previously, we demonstrated that SOX18 expression is increased by shear stress in the pulmonary endothelium. Therefore, in this study, we investigated how mechanical strain mediated through cyclic stretch affects the SOX18/Claudin-5 regulatory axis. Our data demonstrate that SOX18 and Claudin-5 are downregulated in human lung microvascular endothelial cells (HLMVEC) exposed to cyclic stretch and the mouse lung exposed to high tidal mechanical ventilation. Overexpression of SOX18 reduced the loss of Claudin-5 expression in HLMVEC with cyclic stretch and preserved endothelial barrier function. Additionally, overexpression of Claudin-5 in HLMVEC ameliorated barrier dysfunction in HLMVEC exposed to cyclic stretch, although SOX18 expression was not enhanced. Finally, we found that the targeted overexpression of SOX18 in the pulmonary vasculature preserved Claudin-5 expression in the lungs of mice exposed to HTV. This, in turn reduced lung vascular leak, attenuated inflammatory lung injury, and preserved lung function. Together, these data suggest that enhancing SOX18 expression may prove a useful therapy to treat patients with ventilator-induced lung injury.

## Introduction

Mechanical ventilation at high tidal volumes (HTV), which is used to permit gas exchange in patients with acute lung injury (ALI)/acute respiratory distress syndrome (ARDS), can result in ventilator-induced lung injury (VILI) (Bueno et al., 2002; Umbrello et al., 2017). A major feature of VILI is disruption of the endothelial barrier resulting in increased pulmonary vascular permeability and edema (Parker et al., 1984; Dreyfuss et al., 1985; Dreyfuss and Saumon, 1993). The disruption to the lung barrier function in VILI occurs by two main mechanisms, increased lung microvascular pressure from surfactant loss and, more importantly, increased pulmonary alveolar and vascular permeability (Webb and Tierney, 1974; Parker et al., 1984; Parker et al., 1990; De Prost et al., 2011; Mori et al., 2020). Increased vascular permeability leads to edema, which is one of the hallmarks of inflammation. Edema is characterized by increased fluid flux from the vascular system to the interstitial space across the vascular wall. Interestingly, the increase in lung permeability persists after HTV and, in some cases, has been observed to increase, implicating changes in biological function, not just direct physical damage to the alveoli (Egan et al., 1976; Egan, 1980).

SOX-18, a member of the SRY (sex-determining region on the Y chromosome)-related HMG (high-mobility group) box group F family of transcription factors, is an important regulator of vascular permeability and blood vessel development (Dunn et al., 1995; Darby et al., 2001; Zhou et al., 2015). SOX18 has been shown to regulate the expression of an endothelial tight junction protein Claudin-5, which is important in barrier function (Fontijn et al., 2008; Gross et al., 2014; Gross et al., 2018). The claudin family comprises 24 members, each with four transmembrane domains (Tsukita and Furuse, 2000; Lal-Nag and Morin, 2009). Among the claudin family, Claudin-5 is expressed in all organs and is an integral part of the tight junctions of endothelial cells (Morita et al., 1999). The role of SOX18 in the regulation of Claudin-5 is demonstrated by the presence of a conserved SOX-binding site within the promoter of Claudin-5. Silencing of SOX18 and expression of a SOX18 dominant negative mutant significantly decrease the levels of claudin-5, while overexpression of SOX18 significantly increases Claudin-5 levels (Fontijn et al., 2008). We have previously shown that SOX18-mediated increases of Claudin-5 expression protect the lung endothelial barrier against damage from laminar shear stress (Gross et al., 2014). Conversely, we have found that in sepsis, Claudin-5 decreases in a SOX18-dependent manner leading to loss of barrier function and increased permeability (Gross et al., 2018). These findings show that Claudin-5 and SOX18 form a regulatory axis, with SOX18 regulating the expression of Claudin-5, which participates in the formation of tight junctions. The potential role of loss of the SOX18/Claudin-5 regulatory axis in the loss of endothelial barrier function associated with VILI has not been evaluated and was the focus of our investigations.

Our data demonstrate that SOX18 and Claudin-5 are reduced by cyclic stretch applied to cultured human lung microvascular cells (HLMVEC) *in vitro* and HTV in the mouse lung *in vivo*. Further, we found that the overexpression of SOX18 ameliorated the barrier dysfunction associated with cyclic stretch in HLMVEC and reduced inflammatory lung injury, and preserved lung function in the mouse. Thus, our work identifies induction of SOX18 expression as a potential treatment to prevent or diminish VILI in patients undergoing mechanical ventilation.

## Materials and methods

### Cell culture

Primary cultures of human lung microvascular endothelial cells (HLMVECs) were purchased from Cell Applications (San Diego, CA). Cells were cultured in VascuLife medium (Frederick, MD) supplemented VascuLife VEGF-Mv LifeFactors Kit (Frederick, Maryland) and maintained at 37°C in a humidifier with 5% CO_2_.

### Cyclic stretch

For cyclic stretch, 700,000 cells were seeded on six-well BioFlex plates coated with collagen type I (FlexCell Burlington, NC) and maintained in supplemented VascuLife medium for 72 h after confluency, followed by exposure to equibiaxial strain using the FlexCell 5000 Strain Unit. Plates were stretched by applying a vacuum periodically to the bottom of the elastic substrate. The stretch periodic intervals were set at 1 Hz with an 18% amplitude for 8 h as described previously (Quinn et al., 2002). Static control cells were plated under the same conditions on BioFlex plates.

### Adenoviral transduction

HLMVECs were transduced with Adenovirus containing SOX18 (AdSOX18) or Claudin-5 (isoform 1, AdCLDN5) at the indicated MOIs with incubation for 48 h at 37°C before harvesting. Each adenovirus contains an eGFP under its own CMV promoter.

### Western blot analysis

Triton X-100 lysis buffer (containing protease- and phosphatase-inhibitors) was used to lyse lung tissue or cells, as previously described (Catravas et al., 2010). Then, the samples were centrifuged at 20,000 g at 4°C for 20 min and the supernatant was used to calculate the protein concentration by the BCA Protein Assay (Thermo Fisher, Waltham, MA). Tissue and cell extracts (17 μg) were separated using 4–20% Tris-SDS-Glycine PAGE, transferred to Immuno-Blot PVDF membrane by electrophoresis (Bio-Rad Laboratories, Hercules, CA), and then blocked in a Tris-buffered saline solution containing 5% nonfat milk. The membranes were probed with antibodies against SOX18 mouse (Santa Cruz, Dallas, TX) and rabbit (Thermo Fisher, Waltham, MA), Claudin5 mouse (Thermo Fisher, Waltham, MA), pNF-κB pS536 rabbit (Cell Signaling, Danvers, MA), NF-κB rabbit (Cell Signaling, Danvers, MA) and the corresponding secondary antibodies against rabbit and mouse (Thermo Fisher, Waltham, MA). Protein expression was normalized by re-probing with anti-β-actin (Sigma Aldrich, Burlington, MA). Reactive bands were visualized using chemiluminescence (Super Signal West Femto; Pierce, Rockford, IL) on a LI-COR Odyssey image station (Lincoln, NE). Bands were quantified using LI-COR Image Station software.

### Cell imaging

For immunofluorescence experiments, HLMVEC monolayers were trypsinized, then seeded on collagen-covered coverslips, and allowed to attach. The cells were then fixed for 30 min in a solution containing 4% paraformaldehyde (Thermo Fisher, Waltham, MA) and permeabilized for 5 min with 100% prechilled methanol at − 20°C. The cells were incubated for 1 h with 5% BSA, stained with primary antibody overnight at 4°C, then treated for 1 h at room temperature with the corresponding secondary antibody. Lastly, the coverslips with the stained samples were mounted on microscope slides using ProLong Glass Antifade Mountant (Invitrogen, Carlsbad, CA). Immunofluorescent images were observed with a Nikon Eclipse TE2000-U microscope, with Hamamatsu digital camera C11440, and Olympus IX51 microscope with Hamamatsu digital camera C4742–95 was used to capture the immunofluorescent images. ImagePro Plus 7.0 (Casavan and Gaidoukevitch, 2003) or ImageJ software was used to analyze fluorescent intensity. In detail, ImageJ selection tools were used to determine the area of the cell. Then setting measurements were used to include the integrated intensity and area. The fluorescent intensity value of each cell was finalized by deduction of the background intensity. At least 20 cells were measured to obtain the mean value in each observational field, we measured 10 to 42 fields from four independent experiments.

### Ventilator-induced lung injury model

All animal housing protocols were authorized by the institutional animal care and use committee in facilities accredited by the American Association for the Accreditation of Laboratory Animal Care at Augusta University, the University of Arizona, and Florida International University. Adult male C57BL/6NHsd mice (7–8weeks; Harlan) were used in all experiments. The pCMV6-SOX18 or DST-luciferase (control) plasmids were delivered using in vivo-jetPEI reagent *via* the tail vein to specifically target the lung endothelial layer, as described (Aggarwal et al., 2014). After 64 h, the mice were injected in the peritoneum with the anesthetic’s ketamine (100 mg/kg) and xylazine-HCl (10 mg/kg); the anesthetic was supplemented regularly to keep the mice anesthetized during the experimental period. The area around the throat was shaved, and the animals were supine on a heating pad. An incision in the neck midline was made to facilitate endotracheal intubation with a 20 gauge 1in long catheter. The animals were then subjected to mechanical ventilation (Inspira ASVV, model # 55–7058; Harvard Apparatus, Boston, MA) for 8 h with high tidal volumes (HTV; 30 ml/kg) at a rate of 75 breaths per min, a fraction of inspired oxygen concentration (FiO2) of 0.5, an inspiratory to expiratory ratio of 1: 2, and zero end-expiratory pressure (ZEEP). The mice received a single intraperitoneal bolus of 0.8 ml of 0.9% saline upon the initiation of ventilation. Continuous monitoring of end-tidal CO_2_ by a capnograph type 340 (model # 73–3809; Harvard Apparatus) was performed, and an end-tidal CO_2_ of 30–40 mmHg was maintained throughout the experiment. Non-ventilated (NV), sham-operated, control animals were anesthetized and allowed to breathe spontaneously. After the experiment, the mice were euthanized (72 h after plasmid delivery). All lungs were flushed with 4°C EDTA-PBS, removed, snap-frozen in liquid nitrogen, and stored at -80°C until used.

### Assessment of respiratory mechanics

After anesthetizing and performing a neck incision and intubation as described above, a non-invasive oximeter sensor (MouseOx Plus, STARR Life Sciences Corporation, Oakmont, PA, United States) was placed on the neck of the mice as previously described (Aggarwal et al., 2014). Then, animals were connected to a FlexiVent ventilator (Scireq, Montreal, Quebec, Canada), and ventilation was initiated at 10 ml/kg tidal volume with a 150 breaths/min respiratory rate and 2.5 cmH_2_O PEEP. Mice were stabilized for 5 min before measurements commenced. A perturbation sequence including a sinusoidal 1-Hz oscillation was introduced after two lung capacity maneuvers were performed by inflating the lungs to 30 cmH2O. Stepwise increases of the airway pressure to 30 cmH2O followed by decreases in reverse were the dynamic pressure-volume maneuvers performed. Optimal respiratory compliance (ΔV/ΔP) and optimal respiratory elastance (ΔP/ΔV) were calculated from the part of the PV loop during which compliance/elastance is linear. Mice were sacrificed as described above.

### Isolation of bronchoalveolar lavage fluid

PBS (1 ml) was infused and extracted *via* the tracheal cannula, to obtain the Bronchoalveolar lavage fluid (BALF) (Catravas et al., 2010). The BALF was then subjected to centrifugation at 2500 G for 10 min. The supernatant was used to determine protein content using the BCA protein assay or measurement of IL-1β with an IL-1β Mouse ELISA kit (Thermo Fisher, Waltham, MA). The cell pellet was re-suspended in water for 15 s to lyse the red blood cells, and then 20X PBS was added to normalize the salt concentration. The leukocyte cell count was determined using a hemocytometer.

### Measurement of transendothelial electrical resistance

The transendothelial electrical resistance (TER) of HLMVEC monolayers was measured by culturing the cells on gold electrodes with the electrical cell impedance sensor technique, as described (Catravas et al., 2010) using the electrical cell-substrate impedance sensing (ECIS) system (Applied Biophysics, Troy, NY). After 8 h of cyclic stretch, HLMVECs were trypsinized, and 2 × 10^5^ cells/well were immediately plated in eight-well 8W10E ECIS arrays. The ECIS arrays were kept at 37°C and 5% CO_2,_ and the changes in TER were measured using a Z-Theta instrument (Applied Biophysics). For additional experiments, HLMVEC were infected 48 h prior to cyclic stretch with 40 MOI of Ad-SOX18 or 40 MOI of AdCLDN5 and plated onto ECIS arrays as described (Shikata et al., 2005).

### mRNA extraction and quantitative real-time PCR

Frozen mice lung tissues were subjected to cryofracture just prior to isolation procedures. mRNA was isolated from either mouse lung tissues or HLMVECs with the RNeasy Mini Kit Qiagen Sciences, Germantown, Maryland) following the manufacturer’s instructions.

For mRNA quantitation, a NanoDrop spectrophotometer was used, then reverse transcription was performed for 1.5 µg of total RNA with SuperScript VILO Master Mix (ThermoFisher, Waltham, MA). Quantitative real-time PCR (qPCR) was done in triplicate 20 µl reactions using QuantiTect SYBR Green PCR Kit (Qiagen Sciences, Germantown, MD) on an QuantStudio three real-time PCR System (Thermo Fisher, Waltham, MA). Primers specific to the gene of interest were designed using the NCBI tool web-based software for mouse SOX18 and CLDN5 (Primer blast). The SOX18 primer sequence for the forward primer 5′-AAC AAA ATC CGG ATC TGC AC-3′, and the reverse primer is 5′-CGA GGC CGG TAC TTG TAG TT-3’. The CLDN5 primer sequence for the forward primer is 5′-TTT CTT CTA TGC GCA GTT GG-3′, and the reverse primer is 5′-GCA GTT TGG TGC CTA CTT CA-3’. qPCR results were calculated using the comparative CT method as previously described (Schmittgen and Livak, 2008), β_2_ microglobulin was used as a reference gene to compare mRNA expression among samples.

### Statistical analysis

Statistical analysis was performed using the GraphPad Prism software. The mean ± SEM was calculated for all samples. Statistical significance was determined by the unpaired *t*-test (for two groups) or ANOVA (for ≥3 groups) with Newman-Keuls *post hoc* testing for samples with equal standard deviations and Dunnett’s T3 multiple comparisons test for samples with unequal standard deviations. Standard deviations among groups were evaluated with Brown-Forsythe and Bartlett’s tests. A value of *p* < 0.05 was considered significant. Outlier data points were identified by the quartile range method.

## Results

### Cyclic stretch decreases the expression of SOX18 in human lung microvascular endothelial cells (HLMVEC)

**FIGURE 1:**
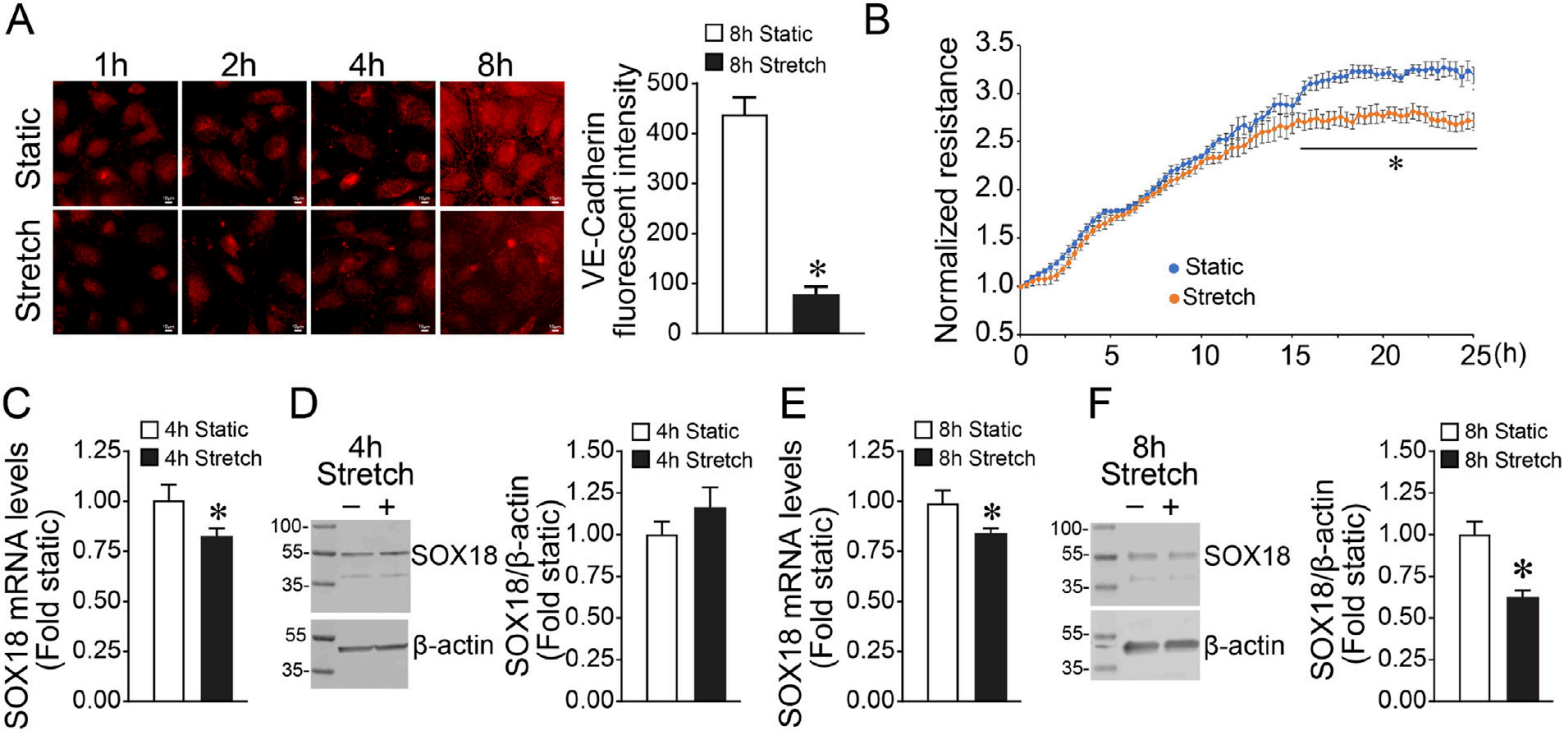
Exposing human lung microvascular endothelial cells to pathologic cyclic stretch reduces Sox18 expression. HLMVECs were analyzed by immunofluorescent staining using a VE-cadherin antibody after being subjected to 18% cyclic stretch for 8 h, replated on coverslips, and fixed at the indicated times. mRNA from HLMVECs were subjected to cDNA synthesis and SYBR green qRT-PCR using specific primers directed to SOX18 sequence and normalized to Beta-2-Microglobulin, a housekeeping gene. Protein extracts prepared from HLMVECs were immunoblotted using SOX18 antibody. VE-cadherin is reduced in the HLMVECs subjected to cyclic stretch **(A)**. Transendothelial resistance recovery is significantly diminished in cells stretched at 18% for 8 h **(B)**. qRT-PCR analysis demonstrated a significant decrease of SOX18 mRNA after cyclic stretch **(C,E)**. Densitometric analysis showed a significant decrease in SOX18 protein levels after 8 h of cyclic stretch **(F)** but not 4 h **(D)**. *p < 0.05 versus Static. N = 10 **(A)**; N = 4 **(B,D)**; N = 5 **(F)**; N = 6 **(C,E)**.

We have previously shown that the angiogenesis-related transcription factor SOX18 plays an important role in preserving endothelial barrier function in response to increased shear stress (Gross et al., 2014). However, its regulation by mechanical strain has not been evaluated. To investigate this response, we subjected HLMVEC to cyclic stretch for 8 h and examined the effects on the endothelial barrier function. Our results show that the levels of VE-cadherin, an important protein in maintaining cell-to-cell adherens junctions, are significantly reduced by cyclic stretch (Figure 1A) and that the barrier recovery is attenuated in HLMVEC exposed to cyclic stretch for 8 h (Figure 1B). Next, we evaluated the impact of cyclic stretch on SOX18 expression. Our results show that while SOX18 mRNA is reduced after 4 h exposure to cyclic stretch (Figure 1C), SOX18 protein levels were unchanged (Figure 1D). However, after 8 h of cyclic stretch, both mRNA (Figure 1E) and protein levels (Figure 1F) were decreased.

### SOX18 overexpression attenuates the loss of barrier function in cultured human lung microvascular endothelial cells exposed to cyclic stretch

**FIGURE 2:**
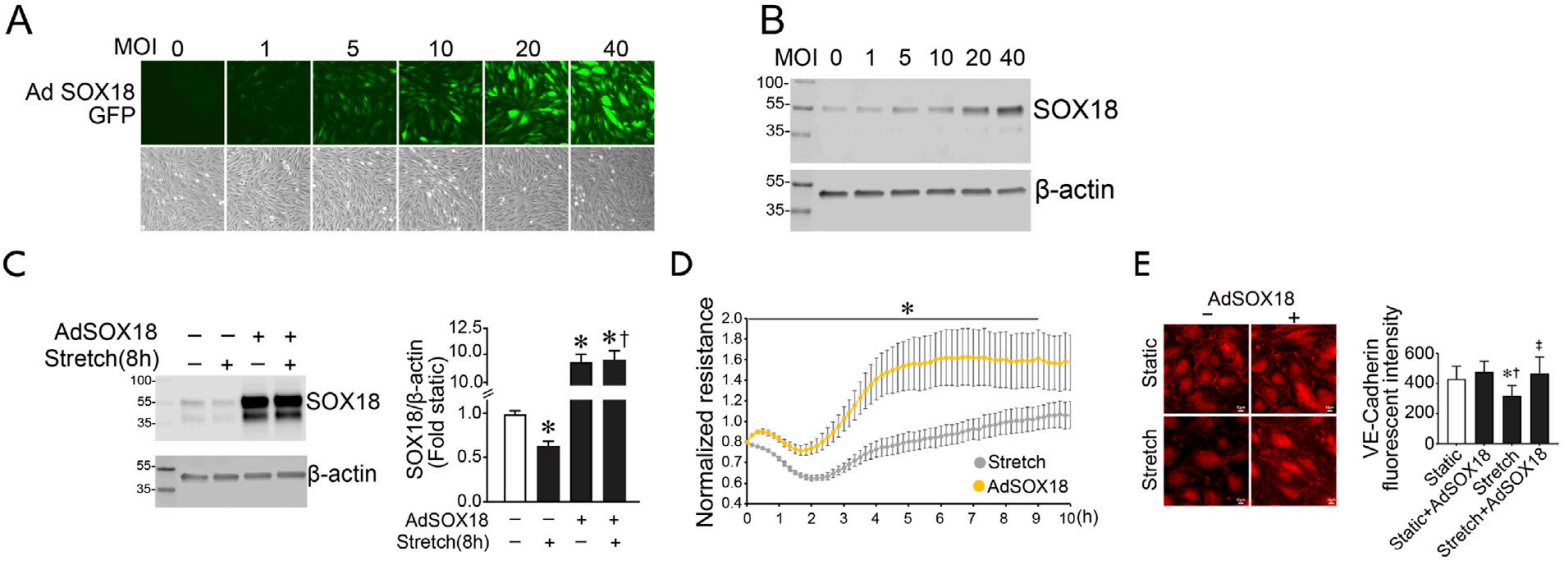
SOX18 overexpression prevents the loss of barrier function in human lung microvascular endothelial cells exposed to cyclic stretch. SOX18 overexpression was induced with an adenovirus (AdSOX18) containing SOX18 and GFP as a reporter (each with its own promoter). HLMVECs were treated with AdSOX18 for 48 h with an MOI of 40 unless otherwise indicated. HLMVEC lysates were analyzed by immunoblot using SOX18 antibody. HLMVECs were analyzed by immunofluorescent staining using VE-cadherin antibody after being subjected to 18% cyclic stretch for 8 h. Fluorescent microscopy shows a proportional increase in adenoviral expression as indicated by the increase in green light fluorescence from the GFP reporter **(A)**. SOX18 overexpression was confirmed by immunoblotting **(B)**. Densitometric analysis shows a significant increase in SOX18 expression after treatment of HLMVECs with AdSOX18 in both static and subjected to cyclic stretch cells relative to the non-adenovirus pretreated cells **(C)**. The transendothelial resistance reduction in HLMVECs subjected to cyclic stretch is ameliorated in cells overexpressing SOX18 **(D)**. Overexpression of SOX18 prevents VE-cadherin loss after cyclic stretch in HLMVECs **(E)**. *p < 0.05 versus Static **(C,E)** or AdSOX18 **(D)**, †*p* < 0.05 versus Stretch **(C)** or Static + AdSOX18 **(E)**, ‡*p* < 0.05 versus Stretch. N = 14–20 **(C)**; N = 4 **(D)**; N = 21–31 **(E)**.

Previously, we have shown that SOX18 overexpression attenuates LPS-induced pulmonary vascular barrier disruption (Gross et al., 2018). Here, we evaluated the effect of SOX18 on the loss of barrier function in response to cyclic stretch. To accomplish this, we utilized an adenovirus containing a human SOX18 cDNA (AdSOX18). Initial studies identified a dose-dependent increase in HLMVEC transduction using the fluorescence of the GFP also present in the virus as a marker (Figure 2A). This correlated with increased SOX18 expression, determined using immunoblot analysis (Figure 2B). An MOI of 40 was utilized for further experiments. AdSOX18 prevented the loss of SOX18 in HLMVEC exposed to cyclic stretch (Figure 2C). Overexpression of SOX18 also improved barrier recovery after cyclic stretch (Figure 2D) and prevented the cyclic stretch mediated loss of VE-cadherin in HLMVEC (Figure 2E).

### Cyclic stretch decreases the expression of Claudin-5 in human lung microvascular endothelial cells (HLMVEC)

**FIGURE 3:**
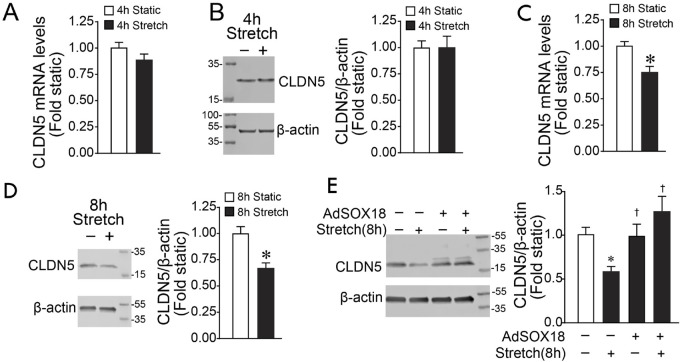
Exposing human lung microvascular endothelial cells to pathologic cyclic stretch reduces Claudin-5 expression. HLMVECs were exposed to 18% cyclic stretch. HLMVEC lysates were analyzed by immunoblot using a specific Claudin-5 antibody. qRT-PCR analysis showed that 4 h of cyclic stretch did not significantly reduce Claudin-5 expression levels **(A)**, which matches the densitometric analysis of Claudin-5 protein levels **(B)**. By contrast, we found a significant decrease of CLDN5 mRNA after 8 hrs of cyclic stretch at 18% **(C)**, reflecting the densitometric analysis showing that Claudin-5 protein levels are significantly decreased **(D)**. Densitometric analysis showed that overexpression of SOX18 prevents the cyclic stretch-dependent Claudin-5 decrease in HLMVECs **(E)**. *p < 0.05 versus Static, †*p* < 0.05 versus Stretch. N = 4 **(B)**; N = 6 **(A,C,D)**; N = 14–20 **(E)**.

Previous research by us and others has demonstrated that SOX18 modulates barrier function through transcriptional regulation of the tight junction protein Claudin-5 (Fontijn et al., 2008; Gross et al., 2014; Gross et al., 2018). Therefore, we investigated if the cyclic stretch-dependent decrease in SOX18 expression leads to a reduced Claudin-5. Unlike SOX18 mRNA levels (Figure 1A), CLDN5 mRNA levels are not reduced by 4 h of cyclic stretch (Figure 3A). Consequently, Claudin-5 protein levels are maintained after 4 h of cyclic stretch (Figure 3B). However, CLDN5 mRNA (Figure 3C) and protein (Figure 3D) were reduced by 8 h of cyclic stretch suggesting the loss of CLDN5 expression occurs after that of SOX18. This is confirmed by our data showing that overexpressing SOX18 prevents the downregulation of Claudin-5 in HLMVEC induced by cyclic stretch (Figure 3E).

### Claudin-5 overexpression alone is sufficient to protect barrier function in HLMVEC exposed to cyclic stretch

**FIGURE 4:**
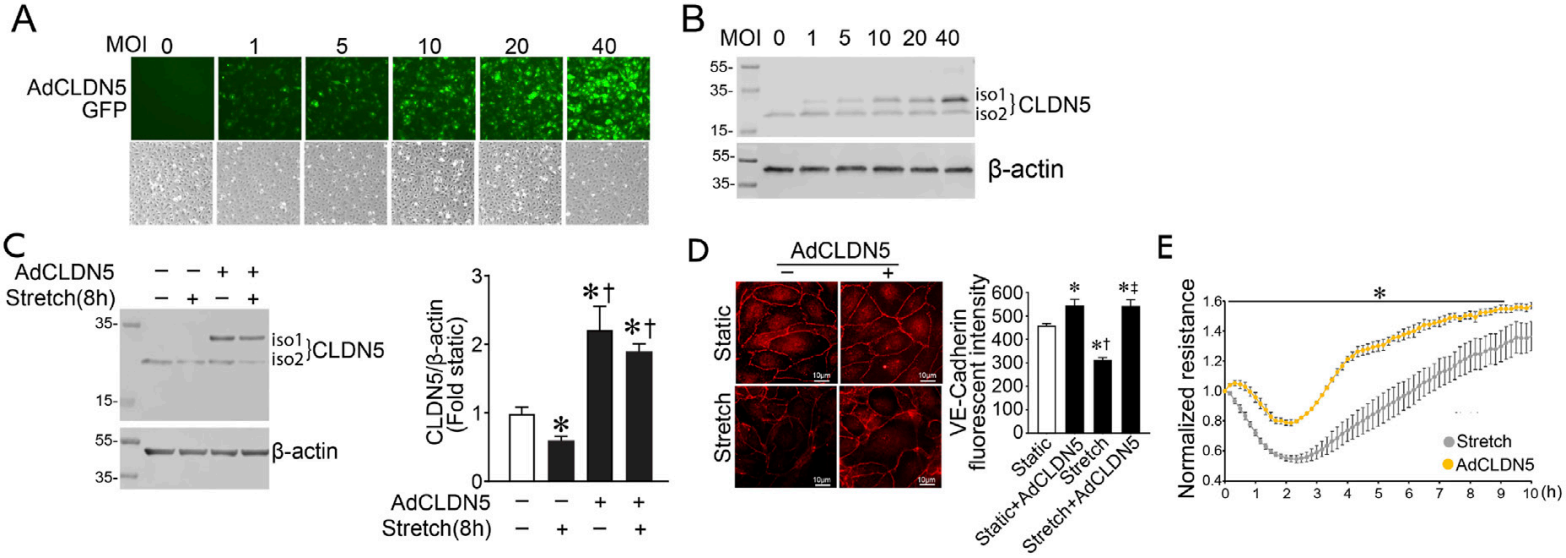
Claudin five overexpression prevents the loss of barrier function in human lung microvascular endothelial cells exposed to cyclic stretch. A CLDN5/GFP coding adenovirus was used to overexpress Claudin-5 isoform 1. HLMVECs were treated with AdCLDN5 for 48 h with an MOI of 40 unless otherwise indicated. HLMVEC lysates were subjected to immunoblot analysis using Claudin-5 antibody. HLMVECs were analyzed by immunofluorescent staining using a VE-cadherin antibody. Treatment of HLMVECs with AdCLDN5 induced a proportional increase in claudin-5 as observed by increased GFP reporter fluorescence **(A)**. Immunoblotting shows a similar proportional increase in Claudin-5 protein levels **(B)**. Total Claudin-5 expression (iso1+iso2) is significantly increased in HLMVECs pretreated with AdCLDN5 and subjected or not to cyclic stretch relative to the non-adenovirus pretreated cells **(C)**. Overexpression of Claudin-5 prevents VE-cadherin loss after cyclic stretch in HLMVECs **(D)**. The transendothelial resistance reduction in HLMVECs subjected to cyclic stretch is ameliorated in cells overexpressing Claudin-5 **(E)**. *p < 0.05 *versus* Static **(C,D)** or AdCLDN5 **(E)**, †*p* < 0.05 *versus* Stretch **(C)** or Static + AdCLDN5 **(D)**, ‡*p* < 0.05 *versus* Stretch. N = 4–6 **(C)**; N = 30–42 **(D)**; N = 3–4 **(E)**.

We next investigated if Claudin-5 is necessary for maintaining barrier function in response to cyclic stretch by over-expressing the protein using a Claudin-5 adenovirus (AdCLDN5) (Figures 4A,B). Initial studies identified a dose-dependent increase in HLMVEC transduction using the fluorescence of the GFP also present in the virus as a marker (Figure 4A). This correlated with increased Claudin-5 isoform 1 (iso 1) expression, determined using immunoblot analysis (Figure 4B). An MOI of 40 was utilized for further experiments. Claudin-5 levels increase with adenovirus transduction and are preserved after cyclic stretch (Figure 4C). Overexpression of Claudin-5 also prevented the cyclic stretch mediated loss of VE-cadherin in HLMVEC (Figure 4D), and this improved barrier recovery after cyclic stretch (Figure 4E).

### SOX18 overexpression preserves Claudin-5 expression and lung function in a mouse model of ventilator-induced lung injury (VILI)

**FIGURE 5:**
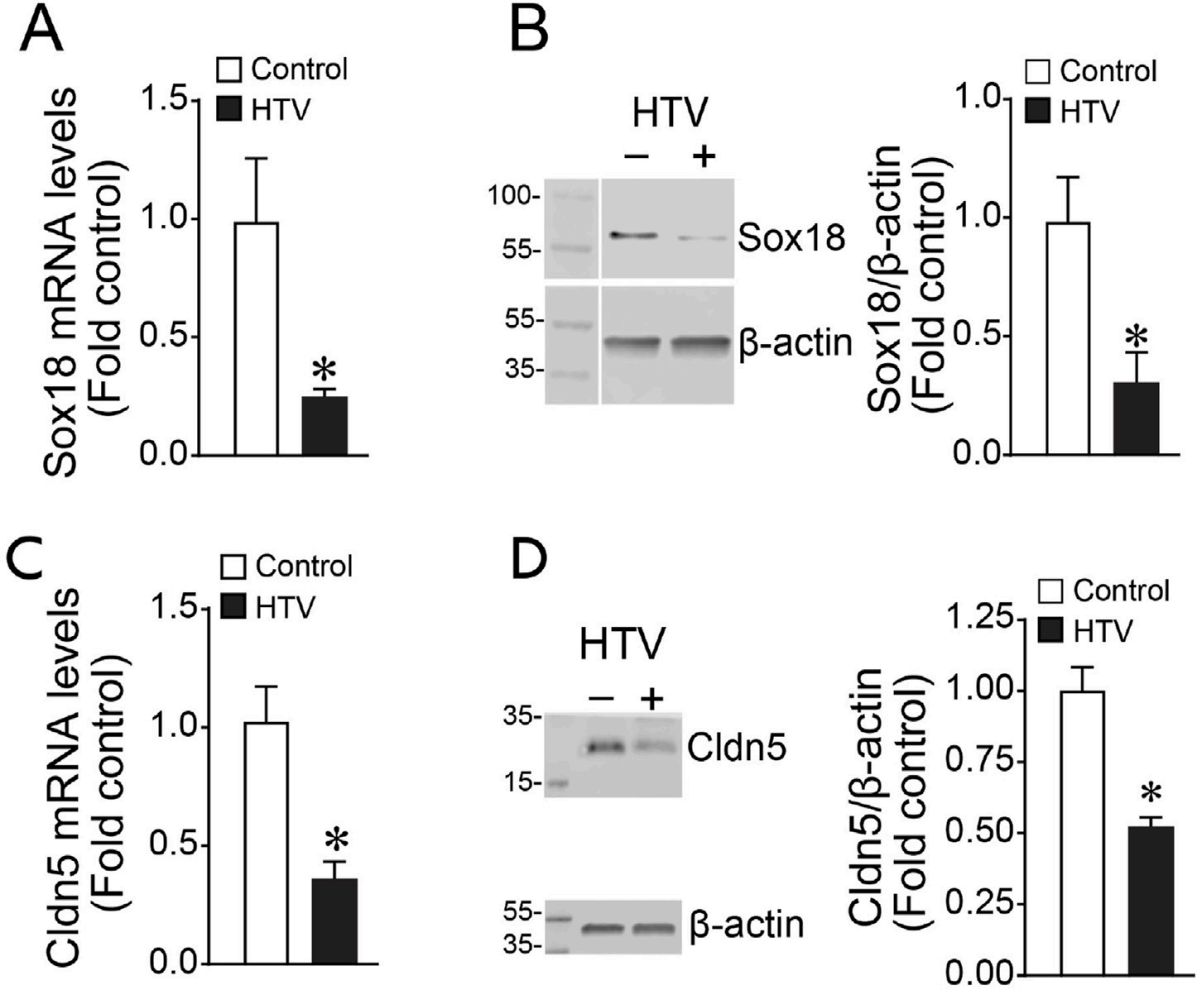
Sox18 and Cldn5 expression are reduced in the mouse lungs subjected to high tidal mechanical ventilation. The mice were exposed or not to ventilation with high tidal volume (HTV; 30 ml/kg, 75 bpm, 0.5 FiO_2_) for 8 h. Protein extracts prepared from lung tissue homogenates were subjected to immunoblot analysis using Sox18 and Claudin-5 antibodies. mRNA isolated from mice lung tissues were subjected to cDNA synthesis and SYBR green qRT-PCR using specific primers directed to Sox18 sequence and normalized to β-2-Microglobulin, a housekeeping gene. qRT-PCR analysis demonstrated a significant decrease of Sox18 expression **(A)**, matching the reduced Sox18 protein levels as determined by densitometric analysis **(B)**. Similarly, Cldn5 mRNA after cyclic stretch is diminished **(C)**, correlating with a decrease in Cldn5 protein levels as measured by immunoblot and densitometric analysis **(D)**. *p < 0.05 versus Control. N = 4–6 **(A)**; N = 3–5 **(B)**; N = 8–12 **(C)**; N = 3–5 **(D)**.

**FIGURE 6:**
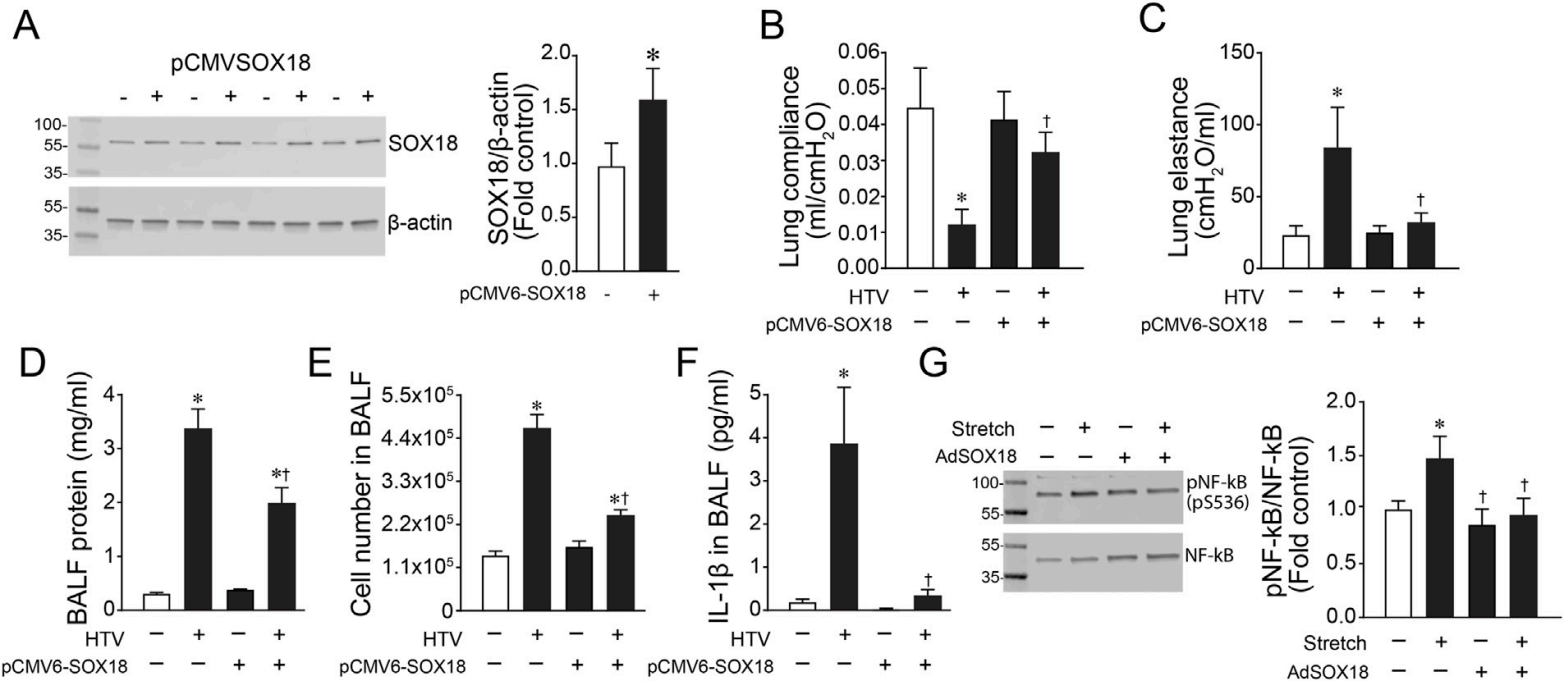
The over-expression of Sox18 preserves lung mechanics in mice exposed to high tidal mechanical ventilation. Mice were injected or not with pCMV6-SOX18 (SOX18) or control pDST-luciferase plasmids using in vivo-jetPEI® *via* the tail vein. After 64 h, the mice were exposed or not to ventilation with high tidal volumes (HTV; 30 ml/kg, 75 bpm, 0.5 FiO2) for 8 h. HLMVECs were treated with AdSOX18 for 48 h to overexpress SOX18 and subjected to cyclic stretch for 8h. Immunoblot analysis demonstrated a significant increase in Sox18 protein levels in the lungs of mice given the pCMV6-SOX18 plasmid **(A)**. The analysis of dynamic pressure-volume relationships in the mouse lung indicated that HTV ventilation caused a loss of Optimal Respiratory System Compliance **(B)** and Respiratory System Elastance **(C)** which is prevented in SOX18 over-expressing mice. SOX18 over-expression attenuated the increase in BALF protein concentration in mice exposed to HTV **(D)**. Total cell count in the bronchoalveolar lavage fluid (BALF) was elevated after HTV exposure, and this was significantly decreased by SOX18 over-expression **(E)**. IL-1β secretion in the BALF was increased with HTV treatment, which was ameliorated by SOX18 overexpression **(F)**. Immunoblotting analysis of HLMVECs showed that overexpression of SOX18 reduced the cyclic stretch-induced increase in pS536-NF-kB **(G)**. *p < 0.05 versus Control, †*p* < 0.05 versus HTV alone. N = 4 **(A)**; N = 3–6 **(B,C)**; N = 6–14 **(D)**; N = 6–12 **(E)**; N = 43–7 **(F)**; N = 7 **(G)**.

To assess the effect of SOX18 and Claudin-5 *in vivo*, we exposed mice to high tidal mechanical ventilation (HTV) to induce VILI. We found that both Sox18 mRNA (Figure 5A) and protein (Figure 5B) levels are decreased with high tidal mechanical ventilation, matching our *in vitro* results. Our results show that high tidal mechanical ventilation also decreases Cldn5 mRNA (Figure 5C) and protein (Figure 5D) in the mouse lung. Using our JetPei-based gene delivery method, we overexpressed SOX18 in the mouse lung (Figure 6A), then exposed mice to HTV. We found that SOX18 over-expression was able to preserve lung function, as demonstrated by the preservation of both optimal respiratory compliance (Figure 6B) and optimal respiratory elastance (Figure 6C). Additionally, the endothelial barrier function was preserved in SOX18 over-expressing lungs as evidenced by a decrease in both protein (Figure 6D) and inflammatory cell (Figure 6E) accumulation in the bronchoalveolar lavage fluid (BALF) in the lungs of mice subjected to high tidal mechanical ventilation. We found that the BALF protein levels were higher than in other studies. This could be due to the 8 h of HTV causing more significant injury to the lung. Consistent with the increased protein and cellular extravasation, the levels of the inflammatory cytokine IL-1β were increased in the BALF, and this was attenuated by SOX18 overexpression (Figure 6F). Correspondingly, phosphorylation of NF-κB at S536, indicative of activation of the pro-inflammatory transcription factor NF-κB, is increased with cyclic stretch in HLMVECs and this is ameliorated by SOX18 overexpression (Figure 6G).

## Discussion

SOX18 is an important regulator of vascular permeability and blood vessel development and is a member of the SRY (sex-determining region on the Y chromosome)-related HMG (high-mobility group) box group F family of transcription factors (Dunn et al., 1995; Darby et al., 2001; Zhou et al., 2015). The importance of SOX18 in blood vessel development has been demonstrated in studies showing that SOX18 mutations result in increased vascular permeability, leading to superficial hemorrhage and severe edema (Pennisi et al., 2000; Irrthum et al., 2003). Additionally, SOX18 is one of two recently identified transcription factors important in regulating blood-brain barrier function (Roudnicky et al., 2020). SOX18 has been shown to regulate the expression of an endothelial tight junction protein Claudin-5, which is important in barrier function (Fontijn et al., 2008; Gross et al., 2014; Gross et al., 2018). The expression profile of Claudin-5 and the other members of the Claudin family changes in several lung diseases associated with edema (Overgaard et al., 2012; Koval, 2013; Gross et al., 2018). The claudin family comprises 24 members, each with four transmembrane domains (Tsukita and Furuse, 2000; Lal-Nag and Morin, 2009). The first claudins, 1 and 2, were identified as integral membrane proteins that localize predominantly in the tight junction strands (Furuse et al., 1998a). Furthermore, transfection of these claudins in L fibroblasts, which lack tight junctions, induced the formation of a tight junction network, showing that claudins are integral parts of the tight junction strands (Furuse et al., 1998b). Among the claudin family, Claudin-5 is expressed in all organs and is an integral part of the tight junctions of endothelial cells (Morita et al., 1999). Our study shows that SOX18 protein and mRNA levels decrease after cyclic stretch in cultured HLMVEC leading to reduced expression of the endothelial tight junction protein, Claudin-5, and impaired barrier function. This finding supports previous work that the SOX18-Claudin-5 axis is important for maintaining endothelial barrier function in the lung (Fontijn et al., 2008; Gross et al., 2014; Gross et al., 2018). While both SOX18 and Claudin-5 overexpression can protect monolayer integrity during cyclic stretch, Claudin-5 overexpression alone is sufficient to protect barrier function implicating Claudin-5 as the effector protein of this function. Additionally, our *in vivo* data showing that SOX18 over-expression attenuates VILI in the mouse lung supports the key role of the SOX18-Claudin-5 axis *in vivo*. It should be noted that a technical limitation in our *in vitro* studies is that our measurements of VE-cadherin and TER could not be performed on the cells directly after stretch. Since *in vitro* cyclic stretch experiments require the use of a plate with an elastic substrate material, obtaining quality pictures for VE-cadherin immunofluorescence is not possible. Further, our TER measurements require using a specific substrate with gold-plated electrodes. Therefore, immunofluorescence and TER analysis required the detachment and replating of the cells in the appropriate substrate for measurements. Thus, our studies evaluated the recovery of the cell junction and barrier function rather than directly measuring the barrier disruption associated with cyclic stretch. EC barrier restoration can be divided into two phases barrier repair and barrier stabilization. As a tight junction protein, Claudin-5 plays a predominant role in barrier stabilization. However, we also observe an increase in barrier repair with SOX18 over-expression, suggesting that SOX18 may play more than one role in barrier recovery after mechanical injury. It should also be noted that differences in recovery rates and the resulting monolayer integrity could also involve differences in cell-cell contact proteins on the EC surface and changes in cell migration. Further studies will be required to investigate this possibility further.

Our data demonstrate that pulmonary endothelial cells do not respond to different mechanical stressors similarly. While cyclic stretch leads to a disruption of barrier function through decreased SOX18 and Claudin-5 expression, we previously demonstrated that shear stress leads to enhanced barrier function through the upregulaton of the SOX18-Claudin-5 axis (Gross et al., 2014). Shear stress is a force that acts parallel to the endothelial cells and results from increased pulmonary blood flow. Therefore, the force is applied to the luminal side of the endothelial cells (Gordon et al., 2020). Consequently, it would be reasonable to conclude that this force would lead to a conformational change in the structure of a mechanosensor with an extracellular domain leading to downstream signaling. Evidence shows this is the case for at least two mechanoreceptors, β1 integrin and Plexin D1, activated by shear stress through a conformational change in their extracellular domains (Xanthis et al., 2019; Mehta et al., 2020). In both cases, the extracellular domain of the mechanoreceptors is in a folded state when there is no flow, and they change to an open state when laminar flow shear stress is applied, leading to downstream signaling (Xanthis et al., 2019; Mehta et al., 2020). Much less is known about the mechanism of mechanoreceptor activation in cyclic stretch. However, as with shear stress, it is likely that mechanosensors respond by undergoing conformational changes specific to the type of mechanical forces active during cyclic stretch. The transient receptor potential (TRP) superfamily of cation channels displays various activation mechanisms and responses, including taste, hearing, touch, and thermal sensation. Among these receptors, evidence suggests that TRPV4 activation is linked to mechanical forces (Loukin et al., 2010). We recently showed that activation of TRPV4 disrupts the endothelial barrier, secondary to a loss of mitochondrial bioenergetics and increased production of the reactive nitrogen species (RNS), peroxynitrite (Lu et al., 2021). ICU patients are treated with mechanical ventilation when they have ARDS (Fan et al., 2017; Walkey et al., 2017; Aoyama et al., 2019; Griffiths et al., 2019; Sklar et al., 2019). We have previously demonstrated that LPS exposure *in vitro* and *in vivo* reduced SOX18 and Claudin-5 expression, leading to loss of barrier function (Gross et al., 2018). This suggests that patients with sepsis-induced ARDS may already be experiencing a decreased expression of SOX18 and Claudin-5 before the initiation of mechanical ventilation. Thus, adding mechanical ventilation may take a double hit to their barrier function, exacerbating the injury to the lung.

Our data also identified high tidal mechanical ventilation-induced inflammatory lung injury as demonstrated by increased release of IL-1β into the BALF. We found that SOX18 over-expression attenuates the high tidal mechanical ventilation-induced IL-1β release in the mouse lung. The expression of IL-1β is stimulated by the pro-inflammatory transcription factor NF-κB (Gross et al., 2018; Wang et al., 2021), and the increase in NF-κB activity induced by pathologic mechanical stress in HLMVECs was also attenuated by SOX18 over-expression. These findings suggest that SOX18 not only reduces lung edema by maintaining barrier function but also has anti-inflammatory activity by inhibiting the NF-κB-induced lung inflammatory response. We recently showed that NF-κB could repress SOX18 transcription *via* an HDAC-mediated epigenetic regulation (Gross et al., 2018; Zemskov et al., 2022). Our current findings suggest that SOX18 attenuates NF-κB activity by attenuating the phosphorylation of the NF-κB subunit p65 at S536. Analysis of the promoter sequence of IκBα, a component of the NF-κB complex, revealed the presence of a SOX18 binding motif 5′-AACAAAG-3′ located 343 base pairs upstream of the ATG start codon. This suggests the possibility that a complex regulatory pathway exists between SOX18 and NF-κB that determines the duration of the endothelial inflammatory response after injury. Future studies will be needed to examine this possibility and to elucidate the mechanism by which SOX18 negatively regulates NF-κB activity.

In this study we have focused on SOX18-mediated regulation of claudin-5. However, other proteins known to regulate claudin-5 expression in cyclic stretch include FoxO1, a transcriptional repressor activated in response to the loss of the adherens junction protein VE-cadherin (Taddei et al., 2008; Beard et al., 2020; Mitra et al., 2021). Similarly, the transcription factor C/EBP-α (CCAAT/enhancer-binding protein-α) positively regulates claudin-5 in response to the adherens junction protein JAM-A (Kakogiannos et al., 2020). Inflammatory signaling can also downregulate the transcription of claudin-5 through the activation of NF-κB and inhibition of the transcription factor ETS-related gene (ERG) (Aveleira et al., 2010; Aslam et al., 2012; Yuan et al., 2012; Clark et al., 2015; Liu et al., 2019). We demonstrate that cyclic stretch reduced VE-cadherin and increased NF-κB activation. As SOX18 ameliorated these effects, it is possible that besides direct binding to the claudin-5 promoter region, SOX18 may also regulate claudin-5 indirectly through the modulation of adherens junctions and inflammatory signaling. However, from our data, it is impossible to separate which effect is dominant. Thus, further studies will be required to investigate this issue.

In addition to its regulatory role in barrier function, SOX18 has been shown to regulate other functions that contribute to the health of the pulmonary vascular endothelial tissue, such as cell growth, migration, and cell viability. For example, SOX18 is important for the angiogenic and lymphagenic process during mouse embryonic development (Darby et al., 2001; François et al., 2008). Similarly, we have previously shown, through microarray analysis, that SOX18 is upregulated in the lungs of an ovine model of congenital heart disease that, results in increased pulmonary blood flow during a period of increased angiogenesis (Tian et al., 2011). Increased expression of SOX18 has also been shown to be important for cell migration in several cancers such as cervical carcinoma, hepatocellular carcinoma (Petrovic et al., 2015; Sun et al., 2019). Similarly, SOX18 can enhance cell migration and proliferation in both *in vitro* and *in vivo* models of prostate cancer by promoting the transcription of the pro-growth factors TCF1, c-Myc, and cyclin D1 (Yin et al., 2017). Additionally, suppressing SOX18 *via* an siRNA has been correlated with impaired cell growth in breast cancer and hepatocellular carcinoma cells (Wang et al., 2015; Zhang et al., 2016). Furthermore, SOX18 also appears necessary for cell survival in hepatocellular carcinoma through regulation of the AMPK/mTOR pathway, as demonstrated by *in vitro* knockdown and overexpression experiments where SOX18 levels negatively correlated with AMPK phosphorylation and positively correlated with mTOR phosphorylation and cell viability (Sun et al., 2019). Together these findings suggest that SOX18 may have other important functions regulating cell growth and/or remodeling in the lung vascular endothelial tissues that could be important for resolving the injury associated with ARDS/VILI. This is important as we have previously shown in a mouse model of sepsis that there is an increase in endothelial cell apoptotic cell death that is directly associated with the disruption of the endothelial barrier and the development of ALI (Gross et al., 2018). Thus maintaining high levels of SOX18 could protect the endothelial barrier from apoptotic cell death and enhance recovery from VILI by facilitating the formation of cell-to-cell connections, inducing cell growth to replenish any cells lost, and increasing cell migration to allow the endothelial cells to fill any remaining gaps in the endothelial barrier. However, further studies will be required to investigate if SOX18 protects the lung against injury through pathways independent of Claudin-5.

In conclusion, our findings demonstrate that the SOX18-Claudin-5 axis is disrupted by VILI and that the over-expression of SOX18 can attenuate the lung injury associated with VILI. These findings suggest that developing pharmacological treatments aimed at either maintaining or increasing SOX18 expression could be useful for treating patients with VILI and with sepsis-induced ARDS that are treated with mechanical ventilation.

## Data Availability

The raw data supporting the conclusions of this article will be made available by the authors, without undue reservation.
